# Cerebral Asymmetries: Complementary and Independent Processes

**DOI:** 10.1371/journal.pone.0009682

**Published:** 2010-03-12

**Authors:** Gjurgjica Badzakova-Trajkov, Isabelle S. Häberling, Reece P. Roberts, Michael C. Corballis

**Affiliations:** Department of Psychology, University of Auckland, Auckland, New Zealand; University of Groningen, The Netherlands

## Abstract

Most people are right-handed and left-cerebrally dominant for speech, leading historically to the general notion of left-hemispheric dominance, and more recently to genetic models proposing a single lateralizing gene. This hypothetical gene can account for higher incidence of right-handers in those with left cerebral dominance for speech. It remains unclear how this dominance relates to the right-cerebral dominance for some nonverbal functions such as spatial or emotional processing. Here we use functional magnetic resonance imaging with a sample of 155 subjects to measure asymmetrical activation induced by speech production in the frontal lobes, by face processing in the temporal lobes, and by spatial processing in the parietal lobes. Left-frontal, right-temporal, and right-parietal dominance were all intercorrelated, suggesting that right-cerebral biases may be at least in part complementary to the left-hemispheric dominance for language. However, handedness and parietal asymmetry for spatial processing were uncorrelated, implying independent lateralizing processes, one producing a leftward bias most closely associated with handedness, and the other a rightward bias most closely associated with spatial attention.

## Introduction

Since the 1860s, when Broca discovered left-cerebral control of speech [1], the left hemisphere has been regarded as dominant, explaining also the fact that most people are right-handed. Since handedness, at least, tends to run in families, a number of theorists have proposed a single two-allele gene, in which one allele codes for left-cerebral dominance and right-handedness, while the other does not specify asymmetry, leaving the directions of handedness and speech dominance to chance [2], [3]. Such models can account for parental influences, as well as the positive but weak correlation between handedness and speech dominance, in which some 95–99% of right-handers and 70–80% of left-handers are left-dominant for speech.

It is also known that most people are right-cerebrally dominant for some nonverbal functions, such as spatial attention and the processing of faces [4], but less is known about how such asymmetries relate to handedness and lateralization of speech. One possibility is that they are achieved by default, as a secondary consequence of left-hemispheric involvement with language [4], [5], so that the greater the left-hemispheric dominance for language the greater the right-hemisphere dominance for nonverbal function. Assuming left-hemisphere dominance is scored positively and right-hemisphere dominance negatively, the correlation between them should be negative. This model has also been referred to as causal complementarity [6]. An alternative is that left-hemispheric dominance is achieved by a pruning of the right hemisphere [2], so that right-hemisphere dominance for processing, whether verbal or nonverbal, is reduced. In this case, then, we might expect the correlation between left-hemisphere dominance for speech and right-hemisphere dominance for spatial processing to be positive.

Empirical studies, though, have suggested that left- and right-hemisphere dominances are largely independent [6], [7]. For example, a study of 270 patients with unilateral brain damage revealed all possible combinations of deficits associated with the lesioned hemisphere, with some showing deficits in both verbal and spatial function, some in verbal or spatial function alone. A small number reversed the usual pattern with spatial but no verbal deficits following left-hemisphere damage, or verbal but no spatial deficit following right-hemisphere damage [6]. The authors concluded that verbal and spatial asymmetries were statistically independent, and that complementary specialization is a statistical norm rather than reflecting a causal relation.

Similar conclusions have been drawn from brain-imaging, using Doppler ultrasonography, in healthy subjects. One study showed atypical patterns of asymmetry for verbal production and spatial attention to be more common in left-handers than in right-handers [8]. In another study, left-hemispheric dominance for verbal production and right-hemispheric lateralization was observed in the majority of 75 right- and left-handed subjects, but around a quarter of them had these functions lateralized in the same hemisphere [7]. The authors concluded that lateralization of cerebral functions depends on independent probabilistic biases, contrary to single-gene models.

To measure cerebral asymmetry, we recorded brain activity using functional magnetic resonance imaging in 155 people during three different tasks. Left-hemispheric lateralization was investigated using a word generation task, and right-hemispheric activation was investigated using two tasks, a landmark task of spatial attention and a face perception task. These tasks were selected because they are known to induce activation in cortical areas that are largely non-overlapping. A word-generation paradigm has been widely used in imaging studies, and has shown very good reliability and concordance with WADA tests [9]–[12]. The landmark task, requiring subjects to determine whether a horizontal line is bisected by a vertical marker, has been consistently shown to engage a right fronto-parietal network in imaging studies [13], [14] and elicit deficits in patients after right-hemisphere stroke [15]. The face perception test involved video clips showing faces producing happy or sad expressions [16]. Although face recognition networks are largely bilateral [17], face processing is considered to be more right-hemisphere dominant, based on deficits in face recognition following unilateral right hemisphere lesion [18] and on brain imaging [19].

To our knowledge, this is the first fMRI study to address the question of how left-hemispheric and right-hemispheric asymmetries, and handedness, might be related.

## Methods

### Participants

Ethics approval was obtained by the Human Ethics Participants Committee at the University of Auckland, New Zealand, and all subjects gave written consent prior to the study. A total of 155 subjects (60 males with a mean age = 23.38 years, SD = 7.09; and 95 females with a mean age = 25.14, SD = 8.43) took part in the study. Many of these were undergraduate students at the University of Auckland. 47 pairs of twins were included in the overall sample and were part of a twin study running concurrently with the current study, but a previous study of over 25,752 individuals revealed no twin-specific or mirroring effects on handedness [20]. All subjects completed a handedness inventory made up of 12 different questions about hand preference (writing, throwing a ball, holding a racquet, lighting a match, cutting with scissors, threading a needle, sweeping with a broom (top hand), shovelling, dealing cards, hammering, holding a toothbrush, unscrewing a lid). They were asked to indicate the hand habitually used for each of these activities by giving two ticks for activities where only one hand is preferred and one tick for each hand when indifferent. Handedness quotients were calculated using the formula 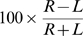 where L and R are the number of ticks allocated for the left and right hands, respectively. Subjects were classified into right-handers and left-handers based on their handedness quotients, with 107 subjects whose quotients were over 0 being considered right-handers and 48 subjects whose quotients were 0 or below being considered left-handers. Writing hand was also considered as a criterion for handedness. All but five subjects were matched on both criteria. The Handedness Inventory score was preferred as it is less confounded with the possibility of switched handedness in subjects. Left-handers were over-represented to ensure reasonable sample sizes in both groups, and to increase the chances of including individuals with atypical patterns of hemispheric lateralization. All 155 subjects performed the word generation task, 154 performed the landmark task, and only 86 performed the faces task. Subjects had no history of neurological or psychiatric disorder and had normal or corrected-to-normal vision.

### Tasks

#### Word Generation Task

Subjects were shown five different letters (F, A, S, B, and M), each projected singly for 30 s onto a screen, and asked to generate covertly as many different words as possible starting with each letter, whilst avoiding proper names and the same words with different endings. Between letter presentations, a fixation cross appeared for 30 s, providing a baseline. The entire experimental run lasted 5 minutes. Prior to scanning, subjects received practice with three different letters (P, R, W), generating words overtly rather than covertly, to ensure instructions were understood.

#### Landmark Task

In the experimental condition, subjects decided whether a horizontal line was bisected exactly in the middle by a small vertical line, and in the control condition they judged whether a bisecting vertical line was present or not. The lines were 5 cm, 8 cm, or 10 cm long, subtending 11, 22 and 33 degrees of visual angle, respectively, and presented black on a white screen for 1 s with an interstimulus interval of 1 s. In the experimental condition the lines were correctly bisected in the middle on 50% of trials, and deviated in 25% of the trials to the left and in 25% of the trials to the right, with biases of 2, 5 or 10% of the lengths of the line. In the control condition the vertical line was presented on half the trials. The different conditions were presented in random order.

During the 1 s inter-stimulus interval, subjects responded by pressing keys with the index finger of the assigned hand for “yes” and the middle finger for “no,” in answer to the questions: “Is the line bisected exactly in the middle?” (experimental condition) or “Is there a vertical mark?” (control condition). Each experimental and control block lasted 30 s and was followed by a 12.5 s baseline that consisted of a black fixation cross. The experiment started with a 5 s introduction screen giving instructions as to which hand to use and which condition should be performed. The following block was repeated 3 times before a new instruction screen appeared. This scheme was repeated 4 times (2 conditions × 2 hands) so that all possible combinations of hand and condition were performed three times by all subjects. The order was counterbalanced. Total scanning time was 8 min 50 s. Prior to the scanning, subjects were given a practice version of the task with 2 bisection and 2 control blocks in order to familiarize them with the task.

#### Faces Task

Subjects were shown video clips of faces (10 male and 10 female) making a happy or sad expression, which served as the experimental condition [16]. The control condition comprised of 40 video clips of moving nonbiological objects (e.g., roulette, blender). A total of 32 experimental blocks (16 with happy/sad expressions and 16 with dynamic object stimuli), each 15 s long, and 33 baseline (fixation cross) blocks, each 10 s long, were presented in a single run (810 s, or ∼14 mins, in total). The order of the blocks was counterbalanced across subjects. Each video clip was presented on a dark background for 1500 ms with 500 ms between clips. Each block was preceded and ended by a 1500 ms gap. The experiment started and ended with a fixation block. One video clip was repeated in each block and the subjects were asked to press a key with their right index finger whenever they saw a repeated video clip. The repeated video clip was never presented immediately following the target video clip.

### Data Acquisition

Images were acquired using a 1.5-tesla Siemens Avanto scanner. T1-weighted structural volume using 3-D MP-RAGE sequence (TR = 11 msec; TE = 4.94 msec; flip angle: 158; FOV: 256×256 mm^2^; up to 176 axial slices, ensuring whole brain coverage, parallel to AC–PC line; slice thickness: 1 mm; interslice gap: 0 mm resulting in 1×1×1 mm voxels) was acquired following the runs for each of tasks. A total of 120 T2*-weighted volumes were acquired during the word generation task, resulting in 60 volumes per condition of interest per subject. A total of 212 T2*-weighted volumes were acquired during the landmark task, corresponding to 48 volumes per condition of interest per subject. A total of 324 T2*-weighted volumes were acquired during the faces task, corresponding to 96 volumes per condition of interest per subject. Two “dummy” scans at the beginning of each run/task were part of the sequence to allow for signal saturation. The EPI acquisition sequence had the following parameters: TR = 2500 msec; TE = 50 msec; flip angle = 90; FOV = 192×192 mm^2^; matrix size: 64×64; 29 slices parallel to AC–PC line; slice thickness: 3 mm; interslicegap: 25% = .8 mm).

### Image pre-processing and analysis

SPM5 software (Wellcome Department of Imaging Neuroscience, London, UK; www.fil.ion.ucl.ac.uk) was used for image processing and analysis. Standard pre-processing steps (realignment, coregistration, normalization and smoothing) were applied. The first volume of the session was used as a reference for realigning the rest of the volumes and a mean of all volumes for the session was created. The T1-weighted structural image was coregistered to the mean of the functional volumes. The structural and functional images were normalized using the normalization parameters estimated with the unified segmentation procedure to the stereotactic coordinate system defined by the MNI. Finally, the functional volumes were spatially smoothed using an anisotropic Gaussian filter of 9×9×9 mm at full-width at half maximum (FWHM). These steps were performed for the three tasks.

For each subject, the pre-processed functional volumes were subject to a 1^st^ level or fixed-effects analysis using the general linear model applied at each voxel across the whole brain. Conditions were modelled by a boxcar waveform convolved with a canonical haemodynamic response function. Movement regressors were also included in the model. Contrast images of interest were also produced (letter vs. baseline for the word generation task; bisection vs. control for the landmark task, and faces vs. objects for the faces task), and imported into a 2^nd^-level or random-effects analysis to obtain group results for each of the tasks. A one-sample t-test was performed on these images to see the general pattern of activation for each of the tasks. The statistical parametric maps were interpreted after applying a family-wise error (FWE) correction with p<.05.

**Figure 1:**
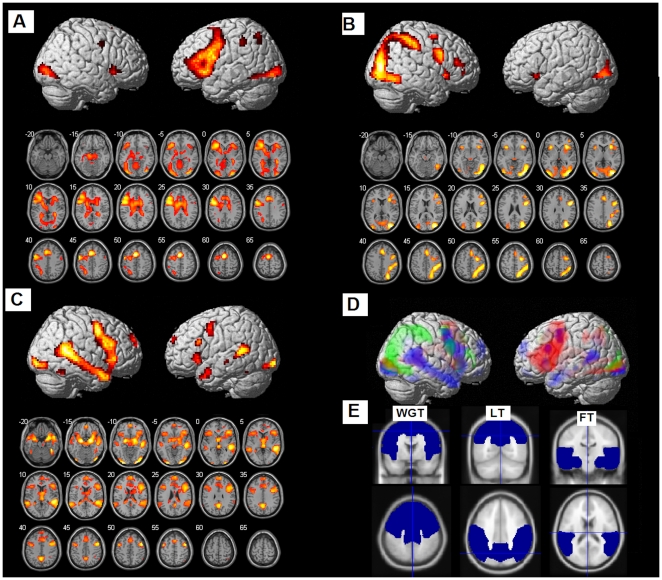
Asymmetrical activation elicited for speech production, spatial processing, and face processing. (A) Group activations from the random effects analysis for the Word Generation Task–WGT. Activations are displayed laterally on a cortical surface rendered brains and through axial slices; (B) Group activations from the random effects analysis for the Landmark Task–LT; (C) Group activations from the random effects analysis for the Faces Task–FT; (D) Activations for the three tasks are shown together on a rendered brain (lateral view) (red = WGT; green = LT; blue = FT); (E) Regions of interest (ROIs) used for calculating the laterality indices for each of the tasks are also shown (top = coronal view; bottom = axial view). Displayed results are significant at p<.05 with family-wise error (FWE) rate correction for multiple comparisons.

Laterality indices were calculated for each subject using the LI toolbox available from the SPM website [21]. This applies a bootstrapping technique allowing about 10 000 indices to be calculated at different thresholds yielding a robust mean, maximum, and minimum index. Taking thresholds into account, an overall weighted bootstrapped laterality index is calculated. Indices range from −1 to +1, with extremes representing complete lateralization to the right and left, respectively. This weighted mean index was calculated for three regions of interest (ROIs) (see Figure 1) that were pre-defined in the LI toolbox [21]; the ROIs were the frontal lobes for word generation, the temporal lobe for the faces task, and the parietal lobes for the landmark task.

## Results

The Statistical Package for the Social Sciences (SPSS, v.17) software was used for all the analyses. An alpha level of *p*<.05 was used for all tests of statistical significance. Two tailed p-values are reported throughout the results section. All post hoc tests were performed with a *Bonferroni* correction.

### Behavioural results

Behavioural data for the word generation task were collected outside the scanner and consisted of the number of words generated during three letter conditions (P, R, W). Analysis of variance was computed to assess the effects of handedness (right-handers vs. left-handers), group (twins vs. nontwins), gender, and letter condition (P, R, W) with the laterality index as a covariate, on task performance. As expected, significantly more words were generated beginning with P (M = 10.04, s.e.m. = .27) than R (M = 8.69, s.e.m. = .25) and W (M = 9.02, s.e.m. = .28) (F _2,292_ = 5.42, *P* = .005). Also, singly-born people (M = 9.69, s.e.m. = .32) performed slightly but significantly better than twins (M = 8.80, s.e.m. = .29) (F _1,146_ = 4.37, *P* = .038).

For the landmark task collected accuracy and RTs (for correct trials only) were recorded during the scanning session. Analysis of variance for accuracy with handedness, group (twins vs. nontwins), gender and condition (bisection vs. control) as factors, and with the laterality index as a covariate, showed significantly higher accuracy in the control condition (M = .94, s.e.m. = .01) than in the bisection condition (M = .85, s.e.m. = .1) (F _1,134_ = 47.62, *P*<.001). A corresponding analysis of RTs showed significantly shorter RTs for the control (M = 549.22, s.e.m. = 7.45) than for the bisection condition (M = 678.81, s.e.m. = 6.91) (F _1,135_ = 391.08, *P*<.001), and significantly shorter RTs for males (M = 597.95, s.e.m. = 10.13) than for females (M = 630.08, s.e.m. = 8.77) (F _1,135_ = 5.74, *P* = .018). No other effects were significant.

For the faces task, accuracy in detecting repetition was recorded during the scanning session. Analysis of variance that assessed the effects of handedness, group (twins vs. nontwins), gender and condition (happy faces, sad faces, objects), and with the laterality index as a covariate, revealed a main effect of condition with better accuracy for the objects (M = .93, s.e.m. = .02) compared to both happy faces (M = .84, s.e.m. = .02) and sad faces (M = .84, s.e.m. = .02) (F _2,128_ = 10.148, *P*<.001). No other effects were significant.

### fMRI results

The group-level activations for each task are shown in Figure 1. Anatomical regions showing significant activation during each of these tasks are presented in Table 1. In brief, for the word generation task, significant activations were observed mostly in the left hemisphere in the supplementary motor area (SMA), inferior frontal gyrus (both pars opercularis and pars triangularis), precentral gyrus, superior and inferior parietal lobules and inferior occipital gyrus. For the landmark task, significant activations were observed mostly in the right hemisphere in the middle and inferior occipital gyrus, inferior frontal gyrus, superior and inferior parietal lobules, supramarginal gyrus, and lingual gyrus. For the faces task, significant activations were observed bilaterally in similar regions, although the level of activation was greater for the right hemisphere as evidenced in the greater T-values. Regions of significant activations included the inferior occipital gyrus, amygdala, middle temporal gyrus, precentral gyrus, fusiform gyrus, inferior frontal gyrus, superior medial gyrus, middle cingulate cortex and SMA.

**Table 1:** Brain regions showing significant activations for each of the tasks.

Brain region	Brodmann area	MNI coordinates			T-value
		x	y	z	
**Word Generation Task**					
*Left hemisphere*					
SMA	6	−3	9	54	20.15
Inferior Frontal Gyrus (p.opercularis)	44	−42	6	27	17.48
Insula	13	−30	24	3	15.01
Inferior Frontal Gyrus (p. triangularis)	45	−45	24	24	13.91
Precentral Gyrus	6	−51	−3	48	13.74
Inferior Occipital Gyrus	18	−36	−84	−9	11.85
Superior Parietal Lobule	7	−27	−63	45	9.19
Inferior Parietal Lobule	40	−42	−39	42	7.78
Cerebellum		−9	−63	−12	6.29
*Right hemisphere*					
Calcarine Gyrus	18	27	−96	0	12.61
Inferior Occipital Gyrus	18	36	−87	−6	11.75
Precentral Gyrus	6	57	−3	42	6.50
**Landmark Task**					
*Left hemisphere*					
Middle Occipital Gyrus	19	−39	−87	−3	7.70
Insula	13	−33	21	−3	7.41
Lingual Gyrus	18	−9	−63	−3	5.11
*Right hemisphere*					
Middle Occipital Gyrus	19	39	−84	9	10.13
Inferior Frontal Gyrus (p.opercularis)	44	48	6	27	9.93
Inferior Occipital Gyrus	18	39	−87	−6	9.95
Superior Occipital Gyrus	19	27	−75	36	9.01
Inferior Parietal Lobule	40	39	−39	45	8.98
Superior Parietal Lobule	7	21	−60	54	8.37
Lingual Gyrus	18	24	−90	−6	8.37
Inferior Temporal Gyrus	20	51	−57	−9	8.20
Supramarginal Gyrus	40	54	−27	45	7.90
Middle Frontal Gyrus	45	48	39	18	5.98
**Faces Task**					
*Left hemisphere*					
Amygdala		−18	−9	−15	12.97
Inferior Occipital Gyrus	18	−24	−99	−9	12.79
Precuneus	31	3	−57	30	9.87
Middle Temporal Gyrus	21	−57	−45	9	8.80
Precentral Gyrus	6	−42	0	57	8.03
Inferior Frontal Gyrus (p. triangularis)	45	−42	18	24	6.73
Superior Medial Gyrus	10	−6	57	27	6.35
SMA	6	0	21	45	5.71
Fusiform Gyrus	37	−42	−54	−21	5.28
*Right hemisphere*					
Inferior Occipital Gyrus	18	30	−96	−9	13.5
Amygdala		21	−6	−15	13.3
Middle Temporal Gyrus	21	54	−39	6	12.04
Precentral Gyrus	6	48	3	51	11.86
Fusiform Gyrus	37	42	−45	−21	10.5
Inferior Frontal Gyrus (p. triangularis)	45	42	27	0	9.54
Superior Medial Gyrus	10	6	60	24	7.96
Middle Cingulate Cortex	32	12	21	39	7.23
SMA	6	3	18	54	6.99

Brodmann area (BA), Montreal Neurological Institute (MNI) coordinates for the peak activation voxel, and T-value are also shown.

Despite the over-representation of left-handers, all three tasks elicited significant overall lateralized activity, as shown by one sample t-tests on the laterality indices, favoring the left hemisphere for word generation (t_154_ = 18.64, *P*<.001), and the right hemisphere for face processing (t_86_ = 9.09, *P*<.001) and landmark (t_153_ = 9.81, *P*<.001) (Fig. 1).

Analyses of variance were computed to assess the effects of handedness, group (twins vs. nontwins), and gender on the laterality indices. Word generation elicited a significantly stronger left-hemispheric bias in right-handers (M = .66, s.e.m. = .04) than in left-handers (M = .31, s.e.m. = .05) (F_1,147_ = 29.98, *P*<.001). The only other significant effect was an interaction between group and gender (F _1,147_ = 8.95, *P* = .003). Simple effects tests revealed that for nontwins, males were more lateralized than females (*P* = .006), but there was no difference between the genders for the twins. The presence of twins had no other significant effects.

Face processing elicited significantly stronger right-hemisphere activity in right-handers (M = −.48, s.e.m. = .07) than in left-handers (M = −.23, s.e.m. = .08) (F_1,79_ = 5.83, *P* = .018). The only other significant effect was an interaction between group and handedness (F _1,79_ = 4.17, *P* = .044). Simple effects tests revealed that for twins, right-handers were more right lateralized than left-handers (*P* = .002), and for singletons the effect did not reach significance. For the landmark task, in contrast, the difference between right-handers (M = −.35, s.e.m. = .05) and left-handers (M = −.32, s.e.m. = .07) was negligible (F_1,146_ = 0.16, *P* = .691). No other effects were significant.

Laterality indices were categorized into those showing left- and right-hemisphere dominance, and the numbers and proportions of right- and left-handers in each dominance category are shown in Table 2. Chi-square tests show that significantly higher proportions of right-handers than left-handers show left-hemisphere dominance during word generation (χ^2^_1, *N* = 155_ = 7.99, *P* = .005), and right-hemisphere dominance for face processing (χ^2^_1, *N* = 76_ = 7.91, *P* = .005). On the landmark task the difference between right and left-handers was negligible (χ^2^_1, *N* = 154_ = 0.01, *P* = .920).

**Table 2:** Number (and percentages) of right- and left-handers with left- and right-hemisphere dominance for each task.

Task	Handedness	Dominant Hemisphere Left	Dominant Hemisphere Right
Word Generation	Right	102 (95.3%)	5 (4.7%)
	Left	39 (81.3%)	9 (18.7%)
Landmark	Right	22 (20.6%)	85 (79.4%)
	Left	10 (21.3%)	37 (78.7%)
Faces	Right	3 (5.7%)	50 (94.3%)
	Left	9 (27.3%)	24 (72.7%)

**Figure 2:**
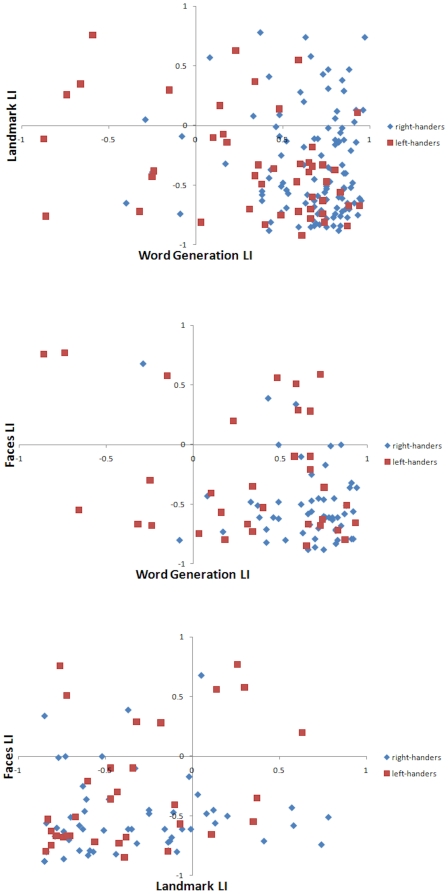
Scatter plots showing the relationships between the three functional asymmetries plotted for each handedness group separately.

The degree of lateralisation on each of the tasks based on handedness (right-handers, left-handers) is visually presented in Figure 2 where three scatter plots show the relationships between the three tasks (word generation and landmark; word generation and faces; landmark and faces). Although all possible patterns of cerebral lateralization were observed, most subjects showed the ‘typical’ cerebral asymmetry pattern, with word generation lateralizing to the left, and landmark and faces lateralizing to the right. A small number of subjects showed a complete reversal of the cerebral asymmetry pattern, and others had both tasks lateralized to same hemisphere.

Table 3 shows the intercorrelations between laterality indices on the three tasks and laterality quotient on the handedness inventory. Again, handedness shows effectively zero correlation with parietal asymmetry induced by the landmark test, but parietal asymmetry nevertheless correlates significantly with frontal asymmetry as elicited by word generation.

**Table 3:** Intercorrelations (2-tailed significance level) between laterality indices and handedness inventory.

	Word Generation	Faces	Landmark	Handedness
Word Generation	*	−.339 (.001)	−.176 (.029)	.357 (.001)
Faces		*	.164 (.131)	−.236 (.028)
Landmark			*	.001 (.991)

The laterality indices for each of the three tasks, writing hand, handedness inventory score, handedness classification based on the handedness inventory score and twin status and pairing are presented for each subject separately in Dataset S1.

## Discussion

First, all three tasks were successful in inducing lateralized activation. Despite the over-representation of left-handers, it is remarkable that all three showed significant overall asymmetry, favouring the left hemisphere for the word generation task and the right hemisphere for the landmark and faces tasks. Second, the areas activated were largely non-overlapping, as is clear from Figure 1. Most prominent activations elicited by the word generation task included the supplementary motor area, inferior frontal gyrus, precentral gyrus, inferior occipital gyrus, and insula in the left hemisphere. The landmark task engaged multiple regions in the occipital, parietal, and frontal lobes in the right hemisphere, supporting earlier findings of the role of a right fronto-parietal network in spatial attention [14]. The faces task showed prominent activations bilaterally in the amygdala, and regions of the temporal, frontal and occipital lobes, but activation was stronger in the right hemisphere, especially in the temporal lobe, which was our region of interest [16].

As expected, right-handers were more likely than left-handers to have word generation lateralized to the left frontal lobe, with figures closely matching those of previous imaging studies [22]–[24]. Right-handers were also significantly more right-cerebrally dominant than left-handers for faces, showing temporal lobe asymmetry, which is consistent with earlier evidence [25]. The lack of significant difference between the handedness groups for the landmark task is consistent with reports of a lack of relationship between handedness and lateralization for spatial processes [7], but is at odds with other studies that have shown such a relationship [26], [27]. The difference may be due to the tasks used, since one of these studies used a mental-rotation task [26] and the other a manual manipulation task [27], whereas the landmark task in our study involved visuospatial judgment.

The significant negative correlations between frontal-lobe asymmetry for word generation and both temporal-lobe asymmetry for face processing and parietal-lobe asymmetry for visuospatial processing implies a complementary relation [28]. More specifically, the right posterior brain regions involved in spatial attention appear to be homologous to Wernicke's area in the left hemisphere [5], [29], implying that the rightward asymmetry is a secondary consequence of the encroachment of language circuits in the left hemisphere. There is no evidence for any asymmetry in spatial attention in animals comparable to that demonstrated by left hemineglect in humans [30]. Similarly, the right-hemispheric bias for the faces task, which displayed emotional expressions, may be complementary to a left-hemispheric bias in the processing of facial speech movements [31]. Indeed, for most of the sample in the current study the word generation task was lateralized to the left, whereas the landmark and the faces tasks were lateralized to the right.

In subjects without a strong bias to left-hemispheric dominance for language, complementarity would be reduced or absent. Genetic theories assume that cerebral asymmetries are driven by a gene in which one allele induces right-handedness and left-cerebral dominance for speech, while the other leaves these asymmetries to chance [2], [3]. In individuals lacking this allele, asymmetries could occur in chance combinations. This might explain the small number of subjects with verbal and nonverbal functions in the same hemisphere, or those who even reverse the normal pattern (see Figure 2).

The pattern of correlations does not conform completely to causal complementarity. In particular, the correlation between handedness and the hemispheric bias on the landmark test was effectively zero, and that between the asymmetries on the faces and landmark tests was nonsignificant. This implies at least some degree of independence, suggesting that at least two lateralizing influences may be operating. One influence is most strongly reflected in handedness and the other in spatial attention. Of the two, handedness may be the more recent in evolutionary time. Its strongest association is with the frontal-lobe asymmetry induced by word generation, perhaps because vocal language itself evolved from manual gesture [32], with the two sharing a common source of lateralization [33]. It has been claimed that chimpanzees do show some tendencies toward right-handedness [34], although the evidence is mixed [35], and some evidence for left-hemispheric dominance for communicative signalling [36], but the combination of right-handedness and left-hemispheric dominance for language is distinctively human.

The right-hemisphere dominance for spatial attention may go back much further in evolution since functional asymmetries have now been widely documented in many species. One possible way of distinguishing different sources of cerebral asymmetry has to do with their possible relation to bodily asymmetries, and in particular to situs inversus of the heart and visceral organs. Handedness does not appear to be reversed in situs inversus [37], but it remains an intriguing possibility that the incidence of situs inversus may be increased with reversed parietal asymmetry.

The nature of the mechanisms underlying cerebral asymmetries remains unclear. A large-scale study suggests that only about a quarter of variation in handedness is due to additive genetic effects, the rest being attributable to environmental influences [20]. No genetic locus has been clearly identified, although some evidence points to the involvement of leucine-rich repeat transmembrane neuronal 1 (LRRTM1) gene on chromosome 2p12, a maternally suppressed gene that appears to be associated paternally with handedness and schizophrenia [38]. This theory has been criticised by Crow et al. [39; but see 40 for counterargument], who has proposed instead that the gene is located in the Xq21.3/Yp11.2 region of homology on the X and Y chromosomes [41]. This possibility, though, is incompatible with genetic polymorphism [42], but possibly compatible with the notion of cerebral asymmetry as a facultative trait with variations due to epigenetic rather than genetic variation [43]. Moreover, it is increasingly suggested that more than one gene is involved [20], [44], and our data lend support to this.

## Supporting Information

Dataset S1 Complete data set with laterality indices on the three tasks, writing hand, handedness inventory score, handedness classification based on the handedness inventory score, and twin status and pairing presented for each subject.
(0.05 MB XLS)
